# Social Media in Pacific Communities: A Scoping Review Exploring Benefits, Challenges and Opportunities for Healthcare

**DOI:** 10.3390/healthcare14121656

**Published:** 2026-06-11

**Authors:** Punipuao Mariner, Samuela ‘Ofanoa, Seita Meneua, Malakai ‘Ofanoa, Felicity Goodyear-Smith, Siobhan Tu’akoi

**Affiliations:** 1 Pacific Health Department, School of Population Health, University of Auckland, Auckland 1023, New Zealand; 2 Department of General Practice and Primary Health Care, University of Auckland, Auckland 1023, New Zealand

**Keywords:** social media, Pacific island people, health promotion, health literacy, education

## Abstract

**Highlights:**

**What are the main findings?**

- Social media is becoming an increasingly popular forum for Pacific people, providing opportunities for connection and access to health information.
- While social media appears to be underutilized for formal health promotion interventions, the few existing studies show positive outcomes on Pacific people’s health knowledge, engagement and behavior.

**What are the implications of the main findings?**

- Culturally grounded social media approaches provide a key opportunity for health promotion and healthcare delivery in Pacific communities.
- There is a need for health professionals and organizations to counter misinformation and support the development of both health and digital literacy skills in the public.

**Abstract:**

Background/Objectives: Despite the potential use of social media for health promotion and education among Pacific communities, there is a lack of understanding regarding how it is used and how interventions can best utilize it. This scoping review aims to explore how social media is used for health purposes within Pacific communities and the potential benefits, challenges and opportunities. Methods: This review followed the PRISMA (Preferred Reporting Items for Systematic Reviews and Meta-Analyses) extension for scoping reviews. Databases and grey literature sources were searched for health-related primary research studies that focused on Pacific communities and included social media. Thematic analysis was used to synthesize the included studies and create codes that reflected the text. Results: A total of 37 studies were included, with most mentioning social media as a key finding (35%), while others approached social media as a recruitment strategy (30%), research topic (22%), intervention tool (14%) or recommendation for use in future approaches (11%). The benefits of social media mentioned in health-related studies for Pacific people include access to health information and services and providing a safe and familiar space. Challenges and risks included misinformation, lack of digital literacy, a disconnect with Pacific cultural norms and the negative impacts of prolonged time on social media. Conclusions: Our findings highlight the potential of culturally grounded social media approaches for health promotion among Pacific communities. To support health promotion in the modern world, there are clear opportunities for developing digital literacy, preserving cultural knowledge, identifying trusted voices to disseminate health knowledge and ensuring social media approaches are evaluated for impact and effectiveness.

## 1. Introduction

Social media has become widely adopted, with users totaling over 5 billion people globally. Platforms such as Facebook, YouTube, WhatsApp, Instagram and TikTok are among the most popular [1]. While traditionally intended for networking, communication and connection, social media has become increasingly influential in healthcare and health promotion, offering accessible and interactive channels for promoting health literacy [1,2]. A systematic review exploring the uses of social media for health purposes found that health institutions use it to share evidence-based health messages, counter misinformation and support behavior change [3]. From the perspective of the public, social media can be a way to exchange social support in online communities to both seek and share health information [3]. For traditionally underrepresented communities who often face disproportionate health burdens, social media has also been highlighted as an opportunity to improve access to healthcare, health education and involvement in research [4,5,6]. Unlike traditional health campaigns that rely on printed materials or in-person outreach, social media can surpass geographic barriers, allowing information to reach large and diverse audiences.

The term ‘Pacific people’ refers to people originating from Pacific Island countries across three sub-regions of Polynesia, Melanesia and Micronesia, which are characterized by diverse cultures, languages and histories [7]. Although each country is distinct, values that are common across Pacific cultures include collectivism, relationality and respect [7]. A significant proportion of Pacific people have migrated from their island countries to larger Western societies such as the United States, Australia and Aotearoa New Zealand (NZ) [8]. Stemming from structural factors, socio-economic disadvantage and barriers to accessing healthcare, Pacific people globally experience inequities across a range of health issues, including cardiovascular diseases, diabetes and mental health conditions [8]. Digital platforms are one way of reducing barriers to healthcare by providing culturally relevant and easily accessible health information, guiding individuals towards prevention, early intervention, and where to get treatment [9]. A cross-sectional survey on 400 Pacific participants in NZ, for example, found that Pacific participants commonly accessed social media and online platforms for health information rather than seeking information from a medical professional [10]. When combined with credible online resources, such as those hosted by government agencies and non-governmental organizations, social media can support informed decision-making and strengthen community participation in health promotion efforts [9]. Although there are diversities across Pacific Island settings related to digital infrastructure, affordability and connectivity [11], social media is an increasingly participated platform for Pacific people. As a result, it presents an important opportunity for Pacific people, who continue to experience health inequities globally.

Despite the potential of social media to improve health promotion and education opportunities for Pacific communities, there is limited evidence on how it is presently utilized in Pacific health research. This gap is important to explore, given that social media usage and its potential impact for health are influenced by contextual and cultural values. This scoping review therefore aims to explore how social media is being used for health purposes in studies with Pacific communities, with a focus on its application across community settings and healthcare initiatives. The specific scoping review questions were:

1. What is known about the uses of social media in health-related studies with Pacific populations?
2. What benefits and challenges have been identified in using social media as a tool for health for Pacific people?
3. What are the opportunities for using social media in health promotion for Pacific communities?

## 2. Materials and Methods

This scoping review followed the Joanna Briggs Institute methodology and used the reporting guidelines offered by the PRISMA (Preferred Reporting Items for Systematic Reviews and Meta-Analyses) extension for Scoping Reviews (checklist located in Supplementary Table S1).

### 2.1. Eligibility Criteria

Health-related studies focused on Pacific populations that utilized or had outcomes related to social media were searched for in this review. The criteria for inclusion or exclusion aligned with the Joanna Briggs Institute criteria of population, concept and context. The population of interest (P) were people with ancestry to any Pacific Island country, including both those living in the Pacific Islands and the diaspora (see Supplementary File S1 for full list). Given the limited research in this area, we included studies on Pacific people that may have also had other ethnic groups included in their sample. However, to ensure that the results and outcomes of studies were primarily reflective of Pacific views and experiences, at least half of participants in the study had to be ethnically Pacific to be included. The concept (C) was any use or outcome related to social media in health-related research. The context (C) included any setting or country globally whereby the research population was Pacific people. Studies published between 2000 and 2025 were eligible for inclusion, given that modern social media platforms emerged in the early 2000s. Primary research studies published as journal articles, reports, theses and conference proceedings were eligible, including qualitative, quantitative and mixed-methods study designs. Literature reviews, content analyses, protocol papers and articles published in languages other than English were excluded.

### 2.2. Information Sources and Search Strategy

Four databases were searched for relevant articles on the 11 December 2025: Embase, Medline (both accessed via Ovid), Scopus and Web of Science. The search strategy for each database included synonyms related to three key concepts: Pacific populations, social media and health. To search for studies with primarily Pacific populations, this concept included search terms such as Pacific, Pasifika, Tonga, Samoa, Tuvalu, Hawaii, Kiribati, Papua New Guinea and Vanuatu. To identify studies that incorporated social media, terms included synonyms such as social network and online social networking, and specific platform names such as Facebook, Instagram, TikTok and YouTube. The health concept included terms related to health and wellbeing. The Boolean operator AND was used between the three key concepts, and OR was used between each synonym within the concept. MESH terms were included where appropriate, and each search was filtered to exclude studies published before 2000.

Grey literature sources were also searched for relevant primary research studies and were assessed for eligibility using the PCC criteria. A Google search using a combination of the keywords “Pacific, “social media” and “health” was undertaken, with the first 50 results reviewed for inclusion. ProQuest was searched using the full keyword strings undertaken for database searches to identify potentially relevant theses and dissertations for inclusion. Health and governmental websites relevant to each Pacific Island country and other countries with large Pacific populations (e.g., NZ, Australia, United States) were searched. Reference lists of the final articles included from the databases were also reviewed for any further relevant studies. Full lists of the database and grey literature sources searched are attached in Supplementary File S1. No formal quality appraisal of studies was undertaken in this review.

### 2.3. Selection of Sources of Evidence

Search results were exported into Covidence (Melbourne, Australia), an online review management tool, and duplicates were removed. The titles and abstracts of search results were screened independently by P.M., S.O. and S.T. Full texts were then retrieved for all the included records, before full-text eligibility assessment between the reviewers was undertaken. Disagreements were discussed between the team of reviewers, with another investigator (F.G.S) available for adjudication if necessary. Articles were excluded if the full text could not be retrieved. An agreement of 91% was reached between reviewers at the title and abstract stage and 95% at full-text stage.

### 2.4. Data Charting and Synthesis

Data items were extracted from full texts by P.M., including the author, year of publication, title, aim, country, study design, details of Pacific people involved, health issue, social media sites identified and uses of social media. Aligned with the objectives of the review to explore the uses, benefits, challenges and opportunities of social media, thematic analysis was used to synthesize the included studies and create codes that reflected the text [12]. Each included study was broadly categorized into the following uses of social media: participant recruitment strategy, intervention, topic being studied, study finding and implications of research. Note that the “study finding” category referred to instances where social media was an independent finding in a study but was not the main topic nor the specific intervention in the study. In order to synthesize and map the available evidence on the potential for social media use in health promotion among Pacific communities, thematic analysis then identified key patterns related to the benefits, challenges and opportunities. ‘Benefits’ were defined as positive contributions of social media to Pacific health; ‘challenges’ encompassed the barriers, risks and adverse consequences of its use and impact; and ‘opportunities’ were defined as actionable strategies or factors that could enhance the effectiveness of social media for Pacific health settings. The charted data presented information from the publications, and no further information was requested from authors.

## 3. Results

### 3.1. Summary of Included Studies

**Figure 1:**
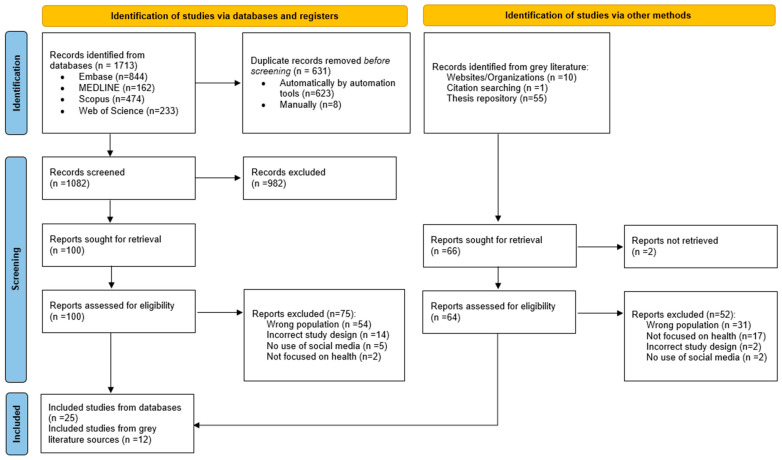
PRISMA diagram.

Figure 1 highlights that 1713 records were identified from database searches, with an additional 66 reports through grey literature searches. A total of 631 duplicate records were removed prior to screening, 623 were removed via the automation tools on Covidence and a further eight articles were manually removed. The titles and abstracts of the remaining 1082 records were then screened independently by reviewers P.M, S.O and S.T, with 982 not meeting the inclusion criteria at this stage. Following this, 166 full texts were sought for retrieval (*n* = 100 from the database search and *n* = 66 by other methods), with two not being accessible. Full text eligibility was then assessed for the remaining records, with reasons for exclusion including wrong population, incorrect study design, no clear use of social media and not clearly health-related. A total of 37 studies were included in this review.

### 3.2. Characteristics of Included Studies

The final 37 studies included 26 peer-reviewed journal articles, seven reports, two theses, and two conference proceedings, all published within the last eight years between 2017 and 2025 (see Table A1 in Appendix A for full details). Studies were conducted across various countries, with seven in both NZ [10,13,14,15,16,17,18] and the United States [19,20,21,22,23,24]; six in Fiji [25,26,27,28,29,30]; three each in Samoa [31,32,33], American Samoa [34,35,36], and Papua New Guinea [37,38,39]; two each in Guam [40,41] and Kiribati [42,43]; and one each in Tonga [44], Vanuatu [45], Palau [46], and the Republic of Marshall Islands [47]. Qualitative methodologies were most common among the studies (41%, *n* = 15). Regarding social media platforms, Facebook was most popular (57%, *n* = 21), followed by Instagram (*n* = 9), YouTube (*n* = 6), Twitter or X (*n* = 5), WhatsApp (*n* = 4), Snapchat (*n* = 2), TikTok (*n* = 2) and LinkedIn, with one instance. An additional 14 studies mentioned social media generally but did not name specific platforms. Health topics covered by the 37 studies were most commonly related to mental health and wellbeing (*n* = 9), sexual and reproductive health (*n* = 7) and non-communicable diseases (*n* = 7).

### 3.3. Uses of Social Media in Studies with Pacific Populations

In health-related studies with Pacific populations, social media was identified as a study finding, a recruitment strategy, the specific topic being studied, an intervention and an implication or recommendation. Most studies (35%, *n* = 13) mentioned social media as a key finding, despite not intentionally researching or focusing on social media in the study aims [10,13,17,18,28,29,34,36,38,39,44,47,48]. Such studies were often qualitative, with Pacific participants discussing the role of social media in accessing health information and promoting health messages related to mental health, sexuality and body image. The second most common use of social media in studies was as a recruitment strategy to engage potential participants (32%, *n* = 12), with platforms such as Facebook, Instagram, WhatsApp and LinkedIn used to distribute online survey links and digital flyers [15,16,19,20,22,24,27,34,36,40,46,48]. For 22% of studies, social media was the specific topic being researched, focusing on how social media shapes health information access, exposure, and user preferences, as well as its impact on health behaviors and lifestyles [14,25,30,31,35,37,41,45]. Only five studies in this review (14%) reported social media as an intervention tool to deliver health promotion messages, educational videos and advocacy campaigns [21,23,26,32,33], including two studies reporting on the same intervention [32,33]. Although direct comparisons of effectiveness were not investigated due to the heterogeneity in study designs and outcome measures, Table 1 summarizes the reported impacts of these social media interventions, which generally showed increased health knowledge and broad public reach. An additional four studies in this review (11%) mentioned social media in the implications, recommending it as a method for future health campaigns and interventions. Studies highlighted social media as a valuable resource for health promotion and social marketing interventions in order to strengthen public health communication, expand reach and ensure the dissemination of accurate health information [17,30,42,43].

### 3.4. Benefits of Social Media in Pacific Health

#### 3.4.1. Accessibility of Health Information and Services

Social media was identified as a way to increase the accessibility of health information and services for Pacific populations. Studies reported social media as an influential health promotion tool that could increase health literacy, promote health services and empower healthy behaviors, with five studies utilizing it as an intervention. For example, a study with Marshallese communities in the United States co-designed educational videos on Type 2 diabetes to be delivered via YouTube [21]. Authors reported that the health education videos provided helpful health information in local languages, and utilizing the YouTube platform ensured access for patients and families to view information on demand, multiple times, and on different devices [21]. Other studies used social media campaigns to promote attendance at vaccination clinics in the community [17], increase awareness of cancer symptoms and diabetes prevention [33,47] and support healthy eating and physical activity challenges [30], in some cases empowering youth to act as influencers and role models [26]. The benefits for more rural or geographically dispersed Pacific populations were also highlighted [28,32,45,47], with social media providing an easy, cost-effective method of both recruiting potential participants from underrepresented communities for research and ensuring broad access to up-to-date health information for communities. The latter was viewed as particularly valuable during health crises, with a qualitative study in Fiji highlighting that disease outbreaks often necessitate open, transparent and rapid communication, and that social media can be a useful informal pathway for this [28].

#### 3.4.2. A Safe and Familiar Space

For many Pacific people, social media is a preferred and familiar space. One study in Vanuatu explored the use of online platforms for health-related purposes among 197 adolescents aged 14–20 years old [45]. The study found that social media platforms like Facebook and TikTok were often used by adolescents as digital tools for searching for and accessing health information. Social media platforms were identified as the preferred space of adolescents to search for health information and follow news stories about health [45]. Other studies identified the benefits of peer interaction and support on social media that allows young people to share personal health experiences, promote culture and see multiple representations of diverse identities, which can help shift attitudes and positively influence health behaviors [20,25,31,36,45]. This was particularly important for health topics that are typically viewed as sensitive or taboo in Pacific cultures [13,17,18,45]. In a study on sexual and reproductive health, for example, Pacific university students in NZ often used social media as a resource for getting information about sexual health topics that may be considered embarrassing or taboo [13]. Tu’i’onetoa and colleagues noted similar findings in Tonga, where young women accessed information on contraception and sexual health topics in privacy by using social media [44]. The authors emphasized a need to ensure information on social media is not only accurate but culturally relevant [44]. Although social media was commonly viewed by studies in this review as a space favored by youth, some reported its popularity across both young and older Pacific generations to keep up with family, community events, culture and Pacific news [10,14,21]. This was further reinforced by eleven studies that utilized social media as a strategy to recruit study participants, recognizing the platforms as common spaces that Pacific communities engage with [15,19,20,22,24,27,34,36,40,46,48].

### 3.5. Challenges and Risks of Utilizing Social Media in Pacific Health

#### 3.5.1. Misinformation and Digital Literacy

A key challenge of the use of social media within the Pacific communities identified was the potential for misinformation, disinformation and conspiracy theories [10,13,14,21,23,25,31,37,38,42,45]. Studies described how the nature of social media enabling easy and rapid dissemination of information could conversely have a negative impact if the information was misleading or false. A report by BBC Media Action on a nationwide survey of 1013 adults in Fiji aged 18+ years found that participants viewed social media and particularly Facebook as a main source of how misinformation in the community spreads [25]. Studies in Papua New Guinea, Samoa and Kiribati similarly identified that misinformation like this may not only confuse people but also lead to low service utilization [42] and threaten health and livelihoods in disease outbreaks [31,37]. In NZ, Pacific community leaders were identified as trusted sources of information for Pacific communities during outbreaks such as COVID-19; however, this could become complicated if leaders were contributing to the spread of misinformation, causing confusion, panic and fear [14]. The need to develop digital literacy skills, particularly for older Pacific generations, was identified to ensure people are able to distinguish what information is trustworthy and what is unreliable [18,20,36,47].

#### 3.5.2. Disconnect with Pacific Cultural Norms

Whilst providing easy access to health information on a global scale, studies in this review outlined a potential disconnect with Pacific cultural values and norms. Differences between Western and Pacific ideals of body image, lifestyle behaviors and sexual health were among the challenges noted [13,16,34,35,36,44]. A qualitative study by Mew and colleagues in American Samoa reported concerns about adolescents being able to maintain their cultural identity due to a “flood of information” about other cultures on social media [36]. The same finding was noted in Tonga, where traditional Tongan norms around young womanhood and sexual behavior are often at odds with the messaging adolescents are exposed to on social media regarding global youth culture [44]. Such conflicts and social pressures could lead to emotional distress, disconnection from family expectations and a weakened sense of belonging. Moreover, participants in one study discussed how Pacific cultural tensions between the older and younger generations may intensify when social media reshapes traditional power dynamics within families [35]. “Fa’asamoa,” or the Samoan way of life, is rooted in Indigenous customs and traditions and is taught and passed down through generations [35]. With increased access to different ideals and norms on social media, older generations felt that traditional fa’asamoa values, such as respect, service and collectivism, were being challenged [35].

#### 3.5.3. Risks Associated with Social Media Usage

Studies in this review also highlighted broader risks associated with social media use among Pacific communities, raising important considerations for its application in healthcare settings. Prolonged time on social media was identified by studies as negatively affecting Pacific young people with regards to mental health, reduced physical activity and exposure to indecent or pornographic material [13,29,35,41,45]. In a qualitative study, adolescents from American Samoa described how a lack of restrictions and filters on social media often resulted in the sharing of explicit and vulgar content [35]. This was similarly highlighted among discussions with Pacific high school students in NZ, who described how exposure to sexually explicit material could occur at very young ages through pop-up advertisements online and on social media [13]. Social pressures and stigma resulting from social media were also described across studies [13,29,30,34,36,44], emphasizing how unrealistic expectations and standards set by social media could result in negative comparisons for Pacific young people. Such findings underscore a need to consider the potential harms of social media for Pacific people’s health, including the increased social pressure, stigma and exposure to unwanted material.

### 3.6. Opportunities for Pacific Social Media Initiatives

**Figure 2:**
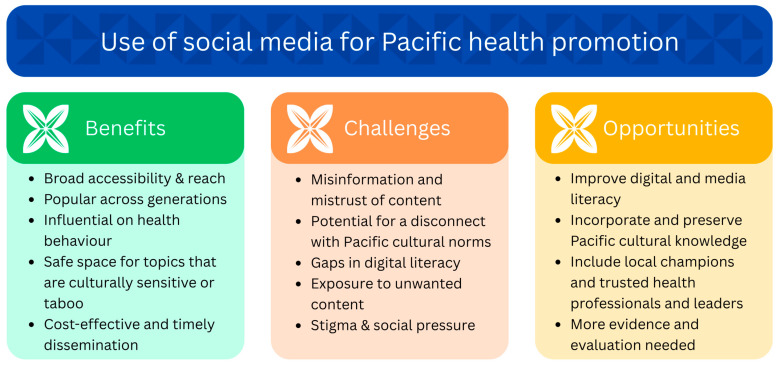
Insights on using social media for Pacific health promotion.

Figure 2 summarizes the benefits and challenges of social media promotion for Pacific communities, alongside key opportunities identified in this scoping review. Recognizing the potential disconnect between social media content and traditional Pacific values, studies in this review emphasized the need for culturally grounded initiatives that incorporate Pacific values and worldviews to ensure health information is shared in a culturally safe and strength-based way [17,31,44]. Suggestions in this review included prioritizing Pacific languages, utilizing storytelling as a form of cultural advocacy and acknowledging the core Pacific value of relationality by focusing approaches on multigenerational families rather than an individual. Prioritizing local champions and trusted Pacific leaders who can share evidence-based health information in formats that are engaging was also deemed important [14,15,20,37,47]. To counter misinformation and inaccurate content, studies in this review reported that content moderation was important, but equally, developing digital and media literacy skills in the population would be critical [14,18,20,25,36,45,47].

## 4. Discussion

This scoping review aimed to explore what is known about social media in health-related studies for Pacific people, including the uses, benefits, challenges and opportunities. Of the 37 final studies included, social media was most commonly mentioned as an independent finding in studies not specifically focused on social media, and as a participant recruitment strategy. This suggests the growing importance of social media among Pacific contexts, in that it is commonly brought up by participants as a factor influencing health and additionally being recognized by researchers as a popular place where Pacific people interact. Despite the recognition of social media’s growing importance in Pacific settings, only a small number of studies in this review utilized it as an intervention tool for health promotion. Such a pattern suggests a disconnect between Pacific people already using and discussing social media for health, while research has only recently begun to test it as a structured health promotion strategy. Helfer and colleagues similarly identified that despite the potential of social media for health promotion, there is limited evidence regarding how it could be used to influence behavior [49]. They identified that health promotion content intended for Aboriginal and Torres Strait Islander people in Australia, who use social media at high rates, needs to prioritize Indigenous cultural views of health and build on existing social capital [49]. Social media–based health promotion approaches that build on the social capital generated by supportive online environments may be more likely to generate greater traction than the confronting and emotion-inducing approaches used in mass media campaigns for some health topics. The gap between communities using and discussing social media and using it as an intervention indicates the need for more Pacific-led social media approaches that are evaluated for effectiveness and impact.

Studies in this review identified that social media is commonly used by Pacific people of all ages to stay connected with family, keep up with local and regional health news and search for health information. Although only a small proportion of studies used social media as a primary intervention for Pacific communities, these interventions generally found positive outcomes related to health knowledge, behavior change and engagement in activities such as vaccination clinics and physical activity challenges. A meta-analysis of 17 studies investigating social media–based mental health interventions (total sample size of *n* = 5624) found similar positive outcomes, showing significant effects for reducing anxiety, depression and stress [50]. Another example in the literature has also shown greater vaccination uptake for an Indigenous Maya community in Guatemala that reported watching culturally and linguistically tailored social media videos addressing COVID-19 misinformation [51]. Such examples link with findings from the current study that outline the considerable potential for Pacific-led social media interventions that are grounded in Pacific culture and worldviews.

Although social media has been acknowledged as being able to spread health information widely and rapidly, the potential for misinformation and conspiracy theories has been identified as a key challenge for health promotion initiatives. This could lead to confusion, mistrust and fear among the community, particularly when trusted leaders share inaccurate information. A cross-sectional survey of 1000 Australian parents with children under five years found that 82.2% used social media for health information for their child [52]. Reasons for engaging with social media before or after a healthcare consultation included the availability of timely, up to date information; exchanging opinions and experiences; and looking for treatment options [52]. The authors reported that it could be challenging for parents to discern the quality of health information on social media, leaving parents open to incorrect information and misinformation [52]. Due to a lack of commitment by social media companies to regulate and moderate their own platforms, Denniss and Lindberg suggest a number of ways to circumvent misinformation, including promoting evidence-based information sharing by public health professionals and developing digital and media literacy education [53]. The increasing popularity of social media across Pacific generations means that building trust and disseminating accurate, evidence-based health information on these platforms is critical.

A strength of this scoping review is that it addresses a key evidence gap regarding how social media is used in health-related contexts for Pacific people on a global scale. This mapping provides valuable insights into current patterns, opportunities and challenges that can influence health promotion strategies and innovative online-based initiatives. The strict inclusion criteria of our scoping review, which required at least 50% of a study’s participants to be of Pacific ethnicity, helped to ensure that findings included in the synthesis were broadly representative of Pacific experiences and perspectives. A potential limitation of this review is that many social media health promotion campaigns exist but remain unevaluated and unpublished in formal peer-reviewed literature. Although grey literature sources were included to ensure reports, theses and conference proceedings could be included, many health promotion campaigns are run on social media without any reporting or evaluation. The findings from this review highlight opportunities for more social media health promotion campaigns in Pacific communities, especially when culturally grounded and community-led, and a need for more evaluation of outcomes and effectiveness. In an increasingly changing digital landscape, it is crucial that Pacific communities are empowered to understand, engage with and be critical of the information they interact with online.

### Implications for Practice

The findings from this review highlight a clear shift in the modern age towards the relevance of digital media for health in Pacific communities. Given that Pacific communities face access barriers to health services, whether it be geographically, financially or culturally, the dissemination of trusted, evidence-based health information through alternative digital platforms is particularly important. Healthcare practitioners and organizations can actively integrate social media into health promotion strategies, partnering with Pacific community groups and leaders to develop culturally grounded content that is relevant, engaging and informative for Pacific people. Building digital literacy should also be prioritized, through community workshops or campaigns that support users to critically assess the credibility of health information. Researchers can support these initiatives with the development of strong, rigorous evaluations to monitor reach, engagement and impacts on understanding and behavior in the long term. At a policy level, governments should continue to strengthen the regulation of online spaces and invest in Pacific-led digital health initiatives that can contribute to addressing health inequities.

## 5. Conclusions

There is widespread use of social media among Pacific people, but it has been utilized as a formal health promotion tool or intervention to only a limited extent. While social media offers significant opportunities to improve health knowledge and engagement in health services, misinformation and mistrust remain major challenges, reinforcing the importance of trusted messengers and digital literacy skills. The findings of this review highlight an opportunity for culturally safe and community-driven social media health promotion approaches to be developed and evaluated. To work towards reducing health inequities for Pacific communities, it is essential that health professionals and organizations meet communities where they are and work together to promote accurate and culturally tailored health information.

## Figures and Tables

**Table 1:** Summary of Pacific social media interventions for health.

Author, Year	Intervention	Participants	Outcomes	Cultural Considerations
Conn, 2021 [26]	A participatory action approach including Talanoa/storytelling and social media training workshops to empower digital promotion of fruit and vegetable businesses and address sustainable food systems and non-communicable diseases in the long term.	*n* = 4 young vegetable and fruit entrepreneurs in Fiji.	Qualitative findings showed that youth entrepreneurs gained better understanding of how to use social media to promote their businesses and the health benefits of their products. Social media also supported networking with other entrepreneurs.	Storytelling is culturally highly valued in the Pacific, and individual champions have a key role to play in advocacy.
McElfish, 2019 [21]	A health education YouTube video focused on blood glucose monitoring and control was developed. An evaluation assessed Marshallese participants’ self-efficacy related to glucometer usage, performing regular blood glucose checks and understanding blood glucose check results.	*n* = 50 Marshallese patients in Arkansas, United States.	Results of the pre-/post-intervention surveys indicated statistically significant increases between participants’ pre-intervention scores and their post-intervention scores on the self-efficacy scale. Statistically significant decreases in A1C were reported among the 20 participants who returned to the clinic for a follow-up visit.	The video was produced in Marshallese with English subtitles, using plain language for those with low health literacy. Participants appreciated the inclusion of a Marshallese physician and community members in the video.
Sofija, 2023 [32] and Spratling, 2018 [33]	The Vave campaign included mass media, social media, printed resources and community outreach to increase awareness of cancer symptoms and early detection. To measure campaign recall, a survey evaluation was conducted.	*n* = 210 community members in Samoa.	The campaign was effective in increasing awareness of cancer and the importance of early detection demonstrated through community recall of campaign messages, increased enquiries to Samoa Cancer Society and improved knowledge. Participants primarily heard about the campaign via television.	Effective relationships in the Samoa community contributed to the success of the campaign.
Tolentino, 2022 [23]	The Next Gen Hawaiʻi social media project utilized social media platforms to spread reliable messaging about COVID-19 and vaccinations.	Native Hawaiian and Pacific Islander communities.	Over 250 messages have been posted, including in-language resources in Chuukese, Chamorro, Marshallese, Samoan, Hawaiian, Ilocano, Tagalog, and other Pacific-basin languages. Reach has included more than 75,000 views on various social media platforms.	Messaging was designed to build individual, community and digital health literacy while integrating core cultural values and strengths of Native Hawaiian and Pacific Islander communities.

## Data Availability

No new data were created or analyzed in this study. Data sharing is not applicable to this article.

## Supplementary Materials

The following supporting information can be downloaded at: https://www.mdpi.com/article/10.3390/healthcare14121656/s1, Table S1: Preferred Reporting Items for Systematic reviews and Meta-Analyses extension for Scoping Reviews (PRISMA-ScR) Checklist [54], File S1: Search strategy.

## Abbreviations

The following abbreviations are used in this manuscript:

NZ	Aotearoa New Zealand
PRISMA	Preferred Reporting Items for Systematic Reviews and Meta-Analyses

## Appendix A

Table A1 presents the characteristics of the final included studies in this review and the key themes.

**Table A1:** Characteristics and themes in reviewed studies.

Author/Year	Country/State	Study Design	Health Topic	Social Media Site(s)	Use of Social Media	Benefits and Opportunities for Social Media in Health	Challenges and Risks for Social Media in Health
Aguon and Kawabata 2023 [40]	Guam	Quantitative	Mental health	WhatsApp, Facebook, Instagram	Social media used to recruit Indigenous Chamoru population.	Social media enabled recruitment of underrepresented ethnic groups. Targeting specific demographics.	None identified.
Amon 2025 [45]	Vanuatu	Mixed methods	Digital health	Facebook, TikTok, YouTube, Instagram, Twitter	The use of online platforms like social media for accessing health information was investigated among adolescents in Vanuatu.	Adolescents seek health information, discuss personal issues, engage with peers and access social support. Rural participants prefer searching social media for health information. Blending traditional cultural norms with the digital environment influences the acceptance and credibility of online health information. Social media can support open, culturally sensitive discussion, improve health literacy and enable dialogue on taboo topics.	This study found that only 24% of participants reported using apps, social media or websites for their health. The limited accessibility of internet infrastructure and socioeconomic disparities can affect digital opportunities. Social media is unregulated which has potential risks of misinformation. Potential for unsolicited unhealthy promotional content. A need for digital literacy skills.
BBC Media Action 2025 [25]	Fiji	Mixed methods	Health in general Mental health	Social media generally	Social media was one of the media types being studied in this report, exploring participants’ access, usage and preferences.	Important information can be shared quickly. Media literacy education can help audiences discern fact from misinformation.	Audiences disengage when they feel content is inaccurate or negative. Risks of misinformation and potential harms for health. A view that social media and Facebook are the main sources through which misinformation spreads.
BBC Media Action 2025 [37]	Papua New Guinea	Mixed methods	Health in general	Facebook, YouTube, WhatsApp, TikTok	Social media was one of the media types being studied in this report, exploring participants’ access, usage and preferences.	Social media is accessible and a key source of news and information. First port of call for information due to speed, immediacy and ad hoc accessibility. Trust in information is contextual and hinges on the source; e.g., health-related news is only trusted when it comes from medical professionals.	Misinformation, unverified or fake accounts and sensational content. Can threaten health and livelihoods when tied to disease outbreaks. Communities with limited or infrequent access to formal media may be more prone to the spread of misinformation via social media.
BBC Media Action 2025 [31]	Samoa	Mixed methods	Health in general	Facebook	Social media was one of the media types being studied in this report, exploring participants’ access, usage and preferences.	Social media was found to be the most popular way of finding information. Participants agreed that sharing information quickly is important. Audiences noted a desire to preserve and promote Samoan culture, share local traditions and support family values.	Less than half of people who identified social media as a popular way of finding information trusted it as a source. Facebook and TikTok were viewed as having fabricated information. Previous experiences of misinformation during epidemics influenced perceptions that social media contributes to false information spreading.
Blas and colleagues 2024 [34]	American Samoa	Qualitative	Mental health	Facebook	Samoan adolescents recruited primarily using a Facebook advertisement and Facebook messenger. Findings identified how social media has been used to communicate bullying, victim blaming and suicidal ideation.	None identified.	Social media can contribute to further stigmatization of mental health issues and social pressure.
Cammock and colleagues 2025 [13]	New Zealand	Qualitative	Sexual and reproductive health	Facebook, Instagram, Twitter, Snapchat	Social media use was a key theme identified by Pacific youth in Talanoa sessions as influencing sexual and reproductive health experiences of Pacific youth.	Social media use was reported as widespread among Pacific youth.	Participants described exposure to sexually explicit material online, in the form of unwanted ads on the internet, pornography and the sharing of compromising images with others. Increasingly, social media was seen as providing unrealistic expectations of what Pacific youth should look like. Misinformation is conveyed frequently, negatively influencing young people.
Conn 2021 [26]	Fiji	Qualitative	Non-communicable diseases	Instagram, Facebook	Social media was used as a research intervention through skills workshops and a health promotion tool for public advocacy.	Social media is crucial to empowering youth as role models, providing a platform to widely disseminate healthy eating messages. Facebook and Instagram present opportunities to widely disseminate advocacy and influence policymakers and society. Entrepreneurs can use digital campaigns to showcase the specific health benefits of ingredients. Traditional forms of marketing have become obsolete as peer groups demonstrate their powerful influence through social media communication.	Training in social media marketing skills is important but not always accessible. While platforms are free, the cost of hiring professional help to promote brands or health messages is often too expensive for youth-led start-ups.
Dalisay 2022 [41]	Guam	Quantitative	Substance use	Facebook, Twitter, Instagram, Snapchat, WhatsApp	Exposure to betel nut–related posts was explored on social media.	Substance-related posts on social media have the potential to shape behaviors through the influence of peer norms. Campaigns aimed at preventing betel nut use may employ social media platforms to disseminate prevention-related messaging.	Early adolescents’ exposure to betel nut–related posts on social media was positively associated with both their susceptibility to use betel nut and reported use. Exposure to substance use–related content on social media could promote substance use.
Desibhatla 2023 [35]	American Samoa	Qualitative	Mental health	Social media generally	Adolescents and key informants in American Samoa were interviewed around the impacts of social media.	Social media can sustain communication between families during a health crisis. Future health interventions that employ types of social media may be made more effective by applying family-mediated approaches to impart health knowledge. The nuanced effects of social media suggest that it may be leveraged as a tool in healthcare with clear regulation.	Mental health burden can be exacerbated. Potential for exposure to and circulation of vulgar content and inappropriate content. Can lead to social isolation. Adults perceive social media as disgracing cultural traditions and practices. Individuals can use fake accounts for misinformation or bullying.
Digital NZ 2022 [14]	New Zealand	Qualitative	COVID-19	Social media generally	Social media was a topic covered in this report on digital inclusion. Findings from participant interviews discussed how social media was used to support dissemination of public health information.	Social media is popular within the Pacific community and is used by the young and old to keep up with family, friends, community and culture, and as a source of Pacific news. Social media is used to quickly connect and bring people together for community events. Social media campaigns helped to get Pacific people to attend pop-up vaccination clinics in the community. Many Pacific people rely on Facebook for information. Opportunity for digital skills training.	Pacific communities were vulnerable to and suffered from misinformation. Pacific people put a lot of trust in their community leaders, but these leaders had sometimes spread misinformation. Online racism was directed at the Pacific community after false information and rumors related to COVID-19 transmission. Many Pacific participants had low trust in online and social media content.
Khan 2023 [27]	Fiji	Quantitative	Mental Health	Facebook, WhatsApp, LinkedIn, Instagram	Snowball sampling on social media platforms such as Facebook, WhatsApp, LinkedIn, and Instagram were used to distribute the online survey.	Social media platforms used for participant recruitment. Social media allowed researchers to distribute an online survey link to a large group, resulting in easy data collection during a period of social restrictions.	Recruitment relied on convenience sampling through social media and so findings may have limited generalizability to the broader Fijian population, particularly those in rural areas or different socio-economic backgrounds.
Marsiglia 2024 [19]	United States	Quantitative	Mental health	Instagram and Facebook	Social media websites such Instagram and Facebook were used to advertise the online survey.	None identified.	None identified.
McElfish 2019 [21]	Arkansas, United States	Mixed methods	Type 2 diabetes	YouTube	YouTube was used as a low-cost platform to deliver a culturally appropriate health education video. The study evaluated its effectiveness for providing evidence-based health information to minority immigrant communities.	YouTube can communicate evidence-based health information to large audiences. Disseminating health education videos via YouTube is an inexpensive means of educating patients in their native language about self-management practices. YouTube videos can be viewed multiple times and on demand, right when patients need the information, without additional cost. Health education videos developed by healthcare providers can provide accurate, timely and cost-effective education and can also be viewed by family who support the patients.	Videos uploaded to YouTube are user-generated, meaning anyone can make a health-related video and post it to the site. Without formal content screening processes, the health information contained in YouTube videos is often misleading or incorrect. Lack of evidence regarding the effectiveness of social media health interventions specifically designed for minority populations.
McElfish 2021 [22]	Arkansas, United States	Quantitative	COVID-19	Facebook	A US Marshallese community Facebook page is used for recruitment and to participate in an online survey.	Social media can be used for rapid recruitment of participants. An effective way to engage with the target population.	With Facebook recruitment, there was a risk of fraudulent responses; a CAPTCHA feature was implemented to address this.
McElfish 2022 [20]	Arkansas, United States	Mixed methods	COVID-19	Facebook	Facebook pages were used to share digital information flyers for recruitment.	The social media pages of local community leaders were one of most trusted sources of public health messages/updates/health information. Platforms like TikTok present opportunities for health engagement and behavior change, such as community-wide physical activity challenges. Social media has a wide reach for participant recruitment.	Digital divide between tech-savvy younger generations and older generations. Older members often struggled with new technologies and platforms like Facebook messenger.
Mew 2024 [36]	American Samoa	Qualitative	Mental health	Facebook	Social media was used for participant recruitment through Facebook advertising. Social media was also discussed by participants as driving cultural change and family conflict by exposing youth to individualistic Western values.	A need to include digital literacy regarding social media and technology in parenting programs. Facebook advertisements and posts can be a good method for participant recruitment.	Social media challenges are a common stressor that places emotional and resource strain on families. Concerns about maintaining cultural identity due to the “flood of information” about other cultures from social media and technology Adolescents feel divided in their cultural identity, not feeling American nor Samoan enough, and many adolescents compare their cultural and language practices to others on social media.
Nelson 2022 [28]	Fiji	Qualitative	Disease outbreaks	Facebook	Social media platforms were used to share and spread messages and information about an outbreak.	Communication during disease outbreaks can become open, transparent, frequent and rapid and utilize informal pathways including social media. Social media also helps informal communication, as it can provide a common platform for engagement.	Social media needs to be used carefully in communicating information about outbreaks, as it can share useful information with a large number of people on health topics such as handwashing but can also create panic and fear.
Odrovakavula and Mohammadnezhad 2021 [29]	Fiji	Qualitative	Wellness	Social media generally	Social media was identified as a factor that influences multiple dimensions of adolescent wellness.	None identified.	Excessive use of social media was identified as having a negative impact on physical, social and spiritual wellness. High-school students who spend more time on social media were less likely to participate in physical activity.
Pilisi 2025 [15]	New Zealand	Quantitative	Burnout	Social media generally	Participants were recruited via social media, with the lead author sharing the survey link across personal social media accounts.	Social media participant recruitment was a key strength of this study. Well-known Pacific community members on social media were included in the steering group of this study, drawing on cultural capital and community experiences.	Recruitment via social media may have missed Pacific individuals who are not active online or those who are more private about these issues.
Puna and Tiatia Seath 2017 [16]	New Zealand	Qualitative	Mental wellbeing	Twitter and Facebook	Social media was used to recruit potential participants.	None identified.	None identified.
Playdon 2022 [48]	Hawaii	Qualitative	Endometrial cancer/sexual and reproductive health	Social media generally	Twitter and Facebook were among some of the recruitment strategies to promote the study and recruit potential participants.	Opportunity for social media as a recruitment strategy to recruit young participants.	None identified.
Rieth 2022 [46]	Palau	Mixed methods	Oral cancer screening	Facebook	The internet-based survey was promoted via Facebook advertisements.	None identified.	None identified.
Rose 2024 [17]	New Zealand	Qualitative	Sexually transmitted diseases	TikTok	Social media was identified as an effective health promotion method for Māori and Pacific youth.	Social media health promotion strategies gain a wide reach. TikTok reaches young people “where they are”. Participants identified the need for strategies to be underpinned by culturally appropriate approaches relevant to their own Māori and Pacific worldviews. Several participants drew on experience with community-led COVID-19 public health initiatives.	None identified.
Schuele 2025 [38]	Papua New Guinea	Qualitative	COVID-19 pandemic	Social media generally	Social media was a source of misinformation and conspiracy theories. Findings show that the spread of misinformation on social media created fear among the public and confused the participants.	Public health researchers need to engage with those larger debates about science, truth and power that have the capacity to undermine evidence-based pandemic strategies.	Social media platforms amplified misinformation and conspiracy theories, contributing to widespread doubts and fear surrounding vaccination. Rural and urban participants in particular were influenced by social media and conspiracy theories.
Sexual Wellbeing Aotearoa 2025 [42]	Kiribati	Mixed methods	Sexual and reproductive health	Social media generally	Implications and recommendations from the research include the need for resourcing social media campaigns.	Social media approaches can improve education and awareness of sexual and reproductive health. Approaches to information sharing are changing. Participants remarked that while the older generation likes community gatherings for information sharing, youth prefer to use modern technology, such as mobile and online social media platforms.	Spread of misinformation resulting in low service utilization.
Singh and Sharma 2022 [30]	Fiji	Quantitative	Obesity	Social media generally	The role of social media was examined by investigating how fitness goal disclosure and viewing others’ weight-loss transformation posts influence healthy lifestyle intentions.	Social marketing interventions can leverage social media to reach customers. Customers sharing experiences, pictures and videos with their network on social media can motivate obese customers to pursue similar goals and adopt a healthier lifestyle. Obese customers who disclosed their fitness goals on social media showed a healthier lifestyle behavior intention when compared to those who did not disclose their goals.	The result confirms that obese customers feel guilt (as opposed to shame) after seeing the weight-loss transformation shared by others on social media.
Sofija 2023 [32]	Samoa	Quantitative	Cancer	Social media generally	Social media was used as a mechanism in the mass media Vave campaign to raise awareness of cancer in Samoa.	To maximize reach, especially among communities with limited access to news media, the campaign adopted a three-pronged community engagement approach including mass media, social media, printed resources and community outreach.	Compared to TV and radio advertisements, social media was a far less commonly recalled medium, perhaps reflecting the cost of telephone and internet usage in Samoa.
Spratling 2018 [33]	Samoa	Quantitative	Cancer	Social media generally	The Vave (Quickly) campaign was a 12-month national social marketing campaign in Samoa comprised of mass and social media coverage, printed resources and community education.	The campaign was successful in increasing awareness of cancer signs and symptoms in the community. Approximately 2000 Samoans (over 1% of the population) received the face-to-face education sessions. Analysis of pre- and post-session questionnaires showed that the sessions were effective in increasing health literacy around cancer signs and symptoms.	None identified.
Sy 2020 [47]	Republic of the Marshall Islands	Qualitative	Non-communicable diseases	Facebook	The study found Facebook and social media to be widely used for personal and public communication; it therefore presents an opportunity for intervention for NCD communication initiatives.	Cost-effective and engaging way of disseminating health materials/education. Communication within and across islands and to other geographic regions to receive information. Participants noted personal examples, stories and testimonies delivered electronically or face-to-face as being the preferred content of health messages. Engaging health champions in media outreach was viewed as an important component in changing habits and preventing and treating diabetes. Increasing support for Facebook-based interventions in delivering evidenced-based prevention messages.	Generational gap in ICT use, with the younger generation having more interest and ability to use various communication and social media platforms than older generations.
Tolentino 2022 [23]	Hawaii, United States	Quantitative	Vaccinations	Facebook, Instagram, YouTube, Twitter, TikTok	The Next Gen Hawaiʻi social media project was initiated in the fall of 2020 to address ongoing public health concerns and the need for reliable and current information across Hawai’i’s diverse communities during the COVID-19 pandemic.	Strength-based social media approach empowered youth to create content for broad reach. Each social media platform has strengths and weaknesses for engagement, as well as specific criteria and algorithms. A strength of social media platforms is that they allow for global engagement and are cost effective. Negative or misleading comments were typically deleted to minimize their impact on viewership and the spread of misinformation.	Visuals are important but time-consuming. While content may be meaningful, it is hard to make a message go viral. A broad reach with open comments enables users who may have not only differing opinions about the public health and health protocols but also various goals to interact publicly. These can include negative comments, problematic links or inappropriate content. In this project, it led to more anti-vaccine and health misinformation being posted in the comments of the boosted posts.
Tu’akoi 2025 [10]	New Zealand	Mixed methods	Rheumatic fever	Social media generally	Social media was identified as a key finding from this study as to where Pacific people access health information.	Social media and the internet were identified as how most people commonly access health information. Popular with both younger and older Pacific generations. Able to share medical journey and experiences. Social media is a good way to access information if you are too shy. Common for health promotion and education, due to its engaging nature and popular use among young people.	Potential for widespread misinformation regarding health, as there are many conspiracy theories.
Tufuga 2025 [24]	United States	Qualitative	Nutrition and diabetes	Social media generally	Pacific Islanders with prediabetes or Type 2 diabetes living in the mainland United States were recruited through email and social media.	Social media websites were used to share a recruitment post promoting participation for PIs living in the Pacific Northwest and California.	None identified.
Tu’i’onetoa 2023 [44]	Tonga	Qualitative	Unplanned pregnancies/sexual and reproductive health	Facebook, YouTube	Social media was discussed by participants as a way that they access information on sexual and reproductive health.	Social media and the internet provided access to information on sexual and reproductive health and contraception for young Tongan women. There is a need for accurate and culturally relevant information, even on social media.	Traditional values and norms around young womanhood and sexual behavior in girls in Tonga can be at odds with the messages on social media and global youth culture.
Wand 2025 [39]	Papua New Guinea	Quantitative	Malnutrition	Facebook, YouTube	This study identified that access to social media outlets such as radio, television and the internet was limited among the participants.	None identified.	Educational status and limited access to social media via radio, television and internet was linked with child malnutrition, which may potentially lead to a lack of understanding, awareness and education about proper nutrition and sanitation.
Whelan 2020 [43]	Kiribati	Mixed methods	Sexual and reproductive health	Social media generally	Recommendation from the research that more youth-friendly information messaging could be developed on social media.	Social media can reach young people via a fresh mode of communication, e.g., Facebook, YouTube videos. Opportunity to work with youth to undertake social media approaches and influence behavior change.	None identified.
Young 2024 [18]	New Zealand	Mixed methods	Sexual and reproductive health	Facebook, Instagram	Participants identified social media as an important resource keeping them informed and up to date with sexual and reproductive information.	Social media platforms such as Facebook and Instagram are some ways that participants learn about sexuality and reproduction. Social media is used as a help-seeking strategy when faced with sexual or reproductive challenges.	The reliability of information on social media can be a challenge.

## References

[1] Kanchan S., Gaidhane A. (2023). Social media role and its impact on public health: A narrative review. Cureus.

[2] Paul B., Headley-Johnson S.-A. (2025). The impact of social media on health behaviors, a systematic review. Healthcare.

[3] Chen J., Wang Y. (2021). Social media use for health purposes: Systematic review. J. Med. Internet Res..

[4] Harry C., Goodday S., Chapman C., Karlin E., Damian A.J., Brooks A., Boch A., Lugo N., McMillan R., Tempero J. (2025). Using social media to engage and enroll underrepresented populations: Longitudinal digital health research. JMIR Form. Res..

[5] Rice E.S., Haynes E., Royce P., Thompson S.C. (2016). Social media and digital technology use among Indigenous young people in Australia: A literature review. Int. J. Equity Health.

[6] Vereen R.N., Kurtzman R., Noar S.M. (2023). Are social media interventions for health behavior change efficacious among populations with health disparities?: A meta-analytic review. Health Commun..

[7] Teariki M.A., Leau E. (2024). Understanding Pacific worldviews: Principles and connections for research. Kōtuitui N. Z. J. Soc. Sci. Online.

[8] Sumibcay J.R.C. (2024). Examining structural racism as the fundamental cause of health inequities among the Indigenous Māori, Native Hawaiian, and Pacific Island peoples in the US and Aotearoa New Zealand: Perspectives from key informant community leaders. SSM Qual. Res. Health.

[9] Gabarron E., Bradway M., Fernandez-Luque L., Chomutare T., Hansen A., Wynn R., Årsand E. (2018). Social media for health promotion in diabetes: Study protocol for a participatory public health intervention design. BMC Health Serv. Res..

[10] Tu’akoi S., Ofanoa M., Ofanoa S., Tohi M., Heather M., Lutui H., Lamont R., Fanueli E., Goodyear-Smith F. (2025). “It Can Hurt Your Heart”: A Co-Designed Cross-Sectional Survey Exploring Pacific People’s Understanding of Rheumatic Fever in Auckland, New Zealand. Healthcare.

[11] United Nations Economic and Social Commission for Asia and the Pacific (2020). Broadband Connectivity in Pacific Islands Countries.

[12] Mak S., Thomas A. (2022). Steps for conducting a scoping review. J. Grad. Med. Educ..

[13] Cammock R., Pousini T., Andrews M., Vaka S., Tautolo E.-S. (2025). Pacific high school students’ experiences of sexual and reproductive health education in Aotearoa New Zealand. Sex. Educ..

[14] Digital N.Z. (2022). Report: Digital Inclusion User Insights—Pacific Peoples.

[15] Pilisi A.S., Sluyter J., Kamu J., Leaumoana S., Sisifa S., Shiu R.N., McCool J. (2025). Burnout Among New Zealand-born Pacific Communities: Results of a Cross-sectional Online Survey. J. Racial Ethn. Health Disparities.

[16] Puna E.T., Tiatia-Seath J. (2017). Defining positive mental wellbeing for New Zealand-born Cook Islands youth. J. Indig. Wellbeing.

[17] Rose S.B., Dunlop A., Gardiner T., Cole M., Garrett S.M., McKinlay E.M. (2024). ‘Every strategy needs to be contributing to erasing the stigma’: Māori and Pacific young people talk about overcoming barriers to testing for sexually transmitted infections. Sex. Health.

[18] Young C.D., Taumoepeau M.M., Hohmann-Marriott B.E., Girling J.E., Bird R.J. (2024). Sexual and reproductive health knowledges: A study with Pacific young people enrolled in an Aotearoa New Zealand tertiary institution. Cult. Health Sex..

[19] Marsiglia S. (2023). Health Access for Native Hawaiians and Pacific Islanders: Latent Determinants of Mental Health Care Service Utilization. Ph.D. Thesis.

[20] McElfish P.A., Felix H.C., Rowland B., Andersen J.A., Willis D.E., Long C.R., Selig J.P., Scott A.J., Kaminicki K.F., Brown A.L. (2022). Impact of COVID-19 on Marshallese Communities in the United States.

[21] McElfish P.A., Rowland B., Riklon S., Aitaoto N., Sinclair K.A., Ima S., Kadlubar S.A., Goulden P.A., Hudson J.S., Mamis S. (2019). Development and evaluation of a blood glucose monitoring YouTube video for Marshallese patients using a community-based participatory research approach. Policy Polit. Nurs. Pract..

[22] McElfish P.A., Willis D.E., Bogulski C., Kelen M., Riklon S., Alik E., Laelan M., Brown A.L., Sinclair K.A., Andersen J.A. (2021). COVID-19 vaccine willingness and hesitancy among Marshallese Pacific Islanders. J. Patient Exp..

[23] Tolentino M., Millerd S., Bali N.Z., Ranido E., Takiguchi J., Atan R., Sentell T. (2022). Next Gen Hawai ‘i: Collaborative COVID-19 social media initiative to engage Native Hawaiian, other Pacific Islander, and Filipino Youth. Hawai’i J. Health Soc. Welf..

[24] Tufuaga D.K., Patten E.V., Gerratt P., Bellini S.G. (2025). Nutrition-Related Experiences and Perceptions of Pacific Islanders with Diabetes in the United States: A Qualitative Study. J. Acad. Nutr. Diet..

[25] BBC Media Action (2025). Fiji: Understanding Audiences and the Role of the Media.

[26] Conn C., Cammock R., Sa’u Lilo L., Nayar S. (2021). Fijian youth entrepreneurs: Championing health through sustainable food systems. Health Promot. Int..

[27] Khan M.G., Patwary M.M., Mamum K.A., Chand A.A., Edward K., Prasad K.A., Browning M.H.E.M., Prasad C., Shuvo F.K. (2023). Prevalence and associated risk factors for mental health problems among young adults in Fiji Island during COVID-19: A cross-sectional study. Front. Public Health.

[28] Nelson S., Abimbola S., Jenkins A., Naivalu K., Negin J. (2022). Information sharing, collaboration, and decision-making during disease outbreaks: The experience of Fiji. J. Decis. Syst..

[29] Odrovakavula L., Mohammadnezhad M. (2021). “Everything else is going to be ok if your spiritual wellness is well”. A qualitative exploration of wellness amongst secondary school students in Fiji. Int. J. Qual. Stud. Health Well-Being.

[30] Singh G., Sharma S. (2022). Obese customers’ fitness goal disclosure on social media: Exploring weight-loss image sharing on emotions and healthy lifestyle aspirations. Eur. J. Mark..

[31] BBC Media Action (2025). Samoa: Understanding Audiences and the Role of the Media.

[32] Sofija E., Harris N., Cuesta-Briand B., Spratling T., Burich S. (2023). The Vave campaign: Impact evaluation of a cancer awareness raising multi-media campaign in Samoa. Health Promot. Int..

[33] Spratling T., Burich S., Cuesta-Briand B., Sofija E. (2018). Increasing community awareness of cancer signs and symptoms in Samoa: The vave campaign. 2018 World Cancer Congress.

[34] Blas V., Mew E.J., Winschel J., Hunt L., Lemusu S.S., Lowe S.R., Naseri J., Toelupe R.L.M., Hawley N.L., McCutchan-Tofaeono J. (2024). Community perspectives on adolescent mental health stigma in American Samoa. PLoS Ment. Health.

[35] Desibhatla M. (2023). Understanding the Current Role of Social Media in American Samoan Adolescent Lives. Master’s Thesis.

[36] Mew E.J., Hunt L., Toelupe R.L., Blas V., Winschel J., Naseri J., Soliai-Lemusu S., Tofaeono J.F., Seui M.A., Ledoux-Sunia T. (2024). O le tagata ma lona aiga, o le tagata ma lona fa’asinomaga (Every person belongs to a family and every family belongs to a person): Development of a parenting framework for adolescent mental wellbeing in American Samoa. Child. Youth Serv. Rev..

[37] BBC Media Action (2025). Papua New Guinea: Understanding Audiences and the Role of the Media.

[38] Schuele E., Toloube O., Anea K., Wohemani R., MacDougall C., Giduthuri J.G. (2025). Learning from community narratives about the COVID-19 pandemic in Papua New Guinea. Health Promot. Int..

[39] Wand H., Naidoo S., Moodley J., Lote N., Toliman P. (2025). Temporal trends and correlates of growth failures in Papua New Guinea children under 5 (2016–2018): Preventing stunting, wasting and underweight. Vulnerab Child. Youth Stud..

[40] Aguon C.T., Kawabata Y. (2023). Examining mental health stigma on Guam: A serial mediation model. Asian Am. J. Psychol..

[41] Dalisay F., Kawabata Y., Buente W., Pokhrel P., Benitez C., Herzog T. (2022). Social media, peer norms, and betel nut susceptibility and use: Evidence from early adolescents in Guam. Front. Commun..

[42] Sexual Wellbeing Aotearoa (2025). Identifying Future Priorities to Improve Sexual and Reproductive Health in Kiribati.

[43] Whelan A. (2020). Kiribati Healthy Families Project: End of Project Evaluation.

[44] Tu’i’onetoa T., Gorman H., McMillan K., Linhart C. (2023). Fofola e Fala kae Talanoa ‘ae Kainga (Roll Out the Mat So That the Family Can Talk): Unplanned Pregnancy and Support for Young Women and Girls in Tonga.

[45] Amon K.L., Wattelez G., Nedjar-Guerre A., Forsyth R., Peralta L.R., Urvoy M.-J., Caillaud C., Galy O. (2025). Exploring the Digital Health Landscape: How adolescents living in urban and rural Vanuatu use social media to access health information. Open Res. Eur..

[46] Rieth K., Dye T., Ikerdu E., McIntosh S., Sy A. (2022). Dental Health Utilization in the Republic of Palau: A Survey to Determine Feasibility of an Oral Cancer Screening Program. The 10th Annual Symposium on Global Cancer Research.

[47] Sy A., Tannis C., McIntosh S., Demment M., Tomeing T., Marriott J., Fukunaga T., Buenconsejo-Lum L., Dye T. (2020). An Assessment of E-health Resources and Readiness in the Republic of the Marshall Islands: Implications for Non-communicable Disease Intervention Development. Hawai’i J. Health Soc. Welf..

[48] Playdon M., Rogers T., Brooks E., Petersen E., Tavake-Pasi F., Lopez J., Quintana X., Aitaoto N., Rogers C.R. (2023). Sociocultural influences on dietary behavior and meal timing among Native Hawaiian and Pacific Islander women at risk of endometrial cancer: A qualitative investigation. Cancer Causes Control.

[49] Hefler M., Kerrigan V., Henryks J., Freeman B., Thomas D.P. (2019). Social media and health information sharing among Australian Indigenous people. Health Promot. Int..

[50] Zhang Q., Huang Z., Sui Y., Lin F.-H., Guan H., Li L., Wang K., Neitzel A. (2025). Social-media-based mental health interventions: Meta-analysis of randomized controlled trials. J. Med. Internet Res..

[51] Miguel L.A., Lopez E., Sanders K., Skinner N.A., Johnston J., Vosburg K.B., Diaz A.K., Diamond-Smith N. (2022). Evaluating the impact of a linguistically and culturally tailored social media ad campaign on COVID-19 vaccine uptake among indigenous populations in Guatemala: A pre/post design intervention study. BMJ Open.

[52] Frey E., Bonfiglioli C., Frawley J. (2023). Parents’ use of social media for health information before and after a consultation with health care professionals: Australian cross-sectional study. JMIR Pediatr. Parent..

[53] Denniss E., Lindberg R. (2025). Social media and the spread of misinformation: Infectious and a threat to public health. Health Promot. Int..

[54] Tricco A.C., Lillie E., Zarin W., O’Brien K.K., Colquhoun H., Levac D., Moher D., Peters M.D., Horsley T., Weeks L. (2018). PRISMA extension for scoping reviews (PRISMA-ScR): Checklist and explanation. Ann. Intern. Med..

