# Supplementary material for: Social Media in Pacific Communities: A Scoping Review Exploring Benefits, Challenges and Opportunities for Healthcare
Source: Healthcare (Basel). 2026 Jun 11;14(12):1656. doi: 10.3390/healthcare14121656 (PMC13299853; doi:10.3390/healthcare14121656)
Supplement: Supplementary file 1 [file healthcare-14-01656-s001.zip › File S1- Search strategy.pdf]

Supplementary file 1: Search strategy

Database searches

Embase

# ▲	Searches	Results	Type
1	health*.mp.	7550357	Advanced
2	(wellbeing or well being or well-being).mp.	285517	Advanced
3	health/	226661	Advanced
4	1 or 2 or 3	7638800	Advanced
5	exp Pacific Islands/	69241	Advanced
6	Pacific*.mp.	80809	Advanced
7	(Pasifika or Pasefika).mp.	342	Advanced
8	"Tonga (people)"/ or Tonga/ or Tonga*.mp.	1128	Advanced
9	Samoa*.mp. or Samoa/	2188	Advanced
10	American Samoa/ or American Samoa*.mp.	662	Advanced
11	Tuvalu/ or Tuvalu*.mp.	143	Advanced
12	Fiji/ or Fiji*.mp.	7204	Advanced
13	Cook islands/ or Cook Island*.mp.	401	Advanced
14	Niue/ or Niue*.mp.	196	Advanced
15	Tokelau/ or Tokelau*.mp.	158	Advanced
16	Kiribati/ or Kiribati*.mp.	384	Advanced
17	Nauru/ or Nauru*.mp.	280	Advanced
18	Marshall Island*.mp. or Marshall Islands/	482	Advanced
19	New Caledonia/ or New Caledonia*.mp.	2259	Advanced
20	Palau/ or Palau*.mp.	828	Advanced
22	Tahiti*.mp.	418	Advanced
23	Hawaii/ or Hawaii*.mp.	16002	Advanced
24	Solomon Islands/ or Solomon Island*.mp.	1277	Advanced
25	Vanuatu/ or Vanuatu*.mp.	1029	Advanced
26	(Wallis and Futuna).mp.	108	Advanced
27	Papua New Guinea/ or Papua New Guinea*.mp.	7501	Advanced
28	PNG.mp.	1960	Advanced
29	French Polynesia/ or French Polynesia*.mp.	1525	Advanced
30	Melanesia/ or Melanesia*.mp.	1617	Advanced
31	"Federated States of Micronesia"/ or Micronesia*.mp.	1658	Advanced
32	Polynesia/ or Polynesia*.mp.	3774	Advanced
33	5 or 6 or 7 or 8 or 9 or 10 or 11 or 12 or 13 or 14 or 15 or 16 or 17 or 18 or 19 or 20 or 21 or 22 or 23 or 24 or 25 or 26 or 27 or 28 or 29 or 30 or 31 or 32	158985	Advanced
34	exp social media/	71265	Advanced
35	social media.mp.	81962	Advanced
36	social networking.mp. or exp social network/	33630	Advanced
37	(facebook or instagram or tiktok or twitter or whatsapp or youtube or reddit or snapchat or pinterest or linkedin).mp. [mp=title, abstract, heading word, drug trade name, original title, device manufacturer, drug manufacturer, device trade name, keyword heading word, floating subheading word, candidate term word]	30484	Advanced
38	34 or 35 or 36 or 37	116630	Advanced
39	4 and 33 and 38	844	Advanced
40	limit 39 to yr="2000 -Current"	844	Advanced

Medline (via Ovid)

# ▲	Searches	Results	Type
1	health*.mp.	5476877	Advanced
2	(wellbeing or well being or well-being).mp.	198396	Advanced
3	1 or 2	5539583	Advanced
4	Pacific*.mp.	60706	Advanced
5	(Pasifika or Pasefika).mp.	231	Advanced
6	Tonga/ or Tonga*.mp.	995	Advanced
7	Samoa*.mp. or Samoa/	1899	Advanced
8	American Samoa/ or American Samoa*.mp.	526	Advanced
9	Tuvalu*.mp.	105	Advanced
10	Fiji/ or Fiji*.mp.	3574	Advanced
11	Cook Island*.mp.	347	Advanced
12	Niue*.mp.	151	Advanced
13	Tokelau*.mp.	131	Advanced
14	Kiribati*.mp.	305	Advanced
15	Nauru*.mp.	223	Advanced
16	Marshall Island*.mp.	415	Advanced
17	New Caledonia/ or New Caledonia*.mp.	2209	Advanced
18	Palau/ or Palau*.mp.	694	Advanced
19	Guam/ or Guam*.mp.	1885	Advanced
20	Tahiti*.mp.	444	Advanced
21	"Native Hawaiian or Other Pacific Islander"/	2077	Advanced
23	Solomon Island*.mp.	1132	Advanced
24	Vanuatu/ or Vanuatu*.mp.	945	Advanced
25	(Wallis and Futuna).mp.	53	Advanced
26	Papua New Guinea/ or Papua New Guinea*.mp.	6338	Advanced
27	PNG.mp.	1521	Advanced
28	French Polynesia*.mp.	1357	Advanced
29	exp Melanesia/ or Melanesia*.mp.	8135	Advanced
30	exp Micronesia/ or Micronesia*.mp.	2916	Advanced
31	exp Polynesia/ or Polynesia*.mp.	13672	Advanced
32	4 or 5 or 6 or 7 or 8 or 9 or 10 or 11 or 12 or 13 or 14 or 15 or 16 or 17 or 18 or 19 or 20 or 21 or 22 or 23 or 24 or 25 or 26 or 27 or 28 or 29 or 30 or 31	91874	Advanced
33	exp Social Media/	21122	Advanced
34	social media.mp.	50118	Advanced
35	social networking/ or online social networking/	6568	Advanced
36	social network.mp.	16239	Advanced
37	(facebook or instagram or tiktok or twitter or whatsapp or youtube or reddit or snapchat or pinterest or linkedin).mp. [mp=title, book title, abstract, original title, name of substance word, subject heading word, floating sub-heading word, keyword heading word, organism supplementary concept word, protocol supplementary concept word, rare disease supplementary concept word, unique identifier, synonyms, population supplementary concept word, anatomy supplementary concept word]	24440	Advanced
38	33 or 34 or 35 or 36 or 37	76418	Advanced
39	3 and 32 and 38	164	Advanced
40	limit 39 to yr="2000 -Current"	162	Advanced

Scopus

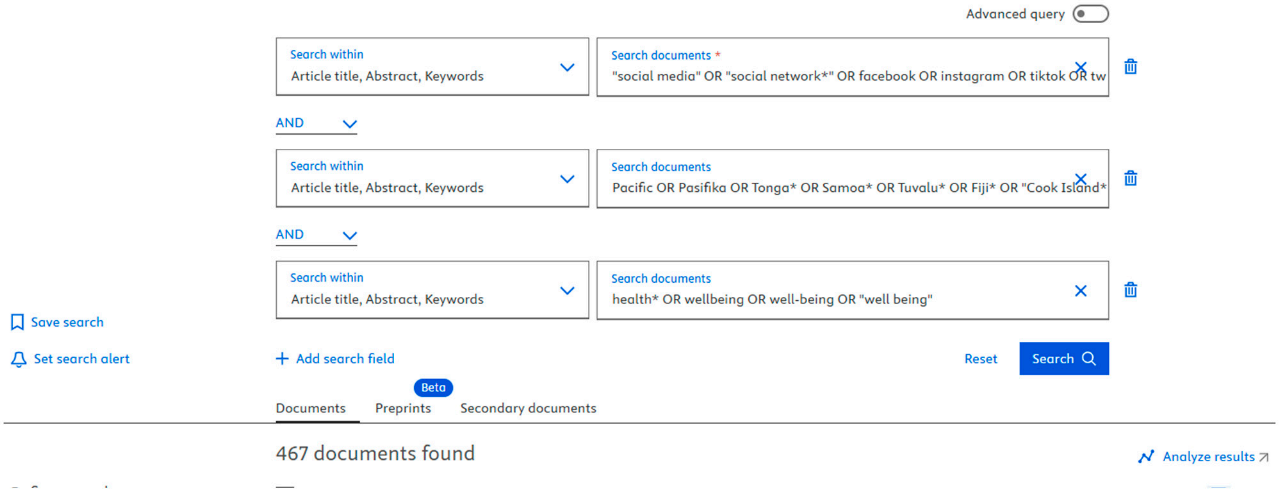

Web of Science

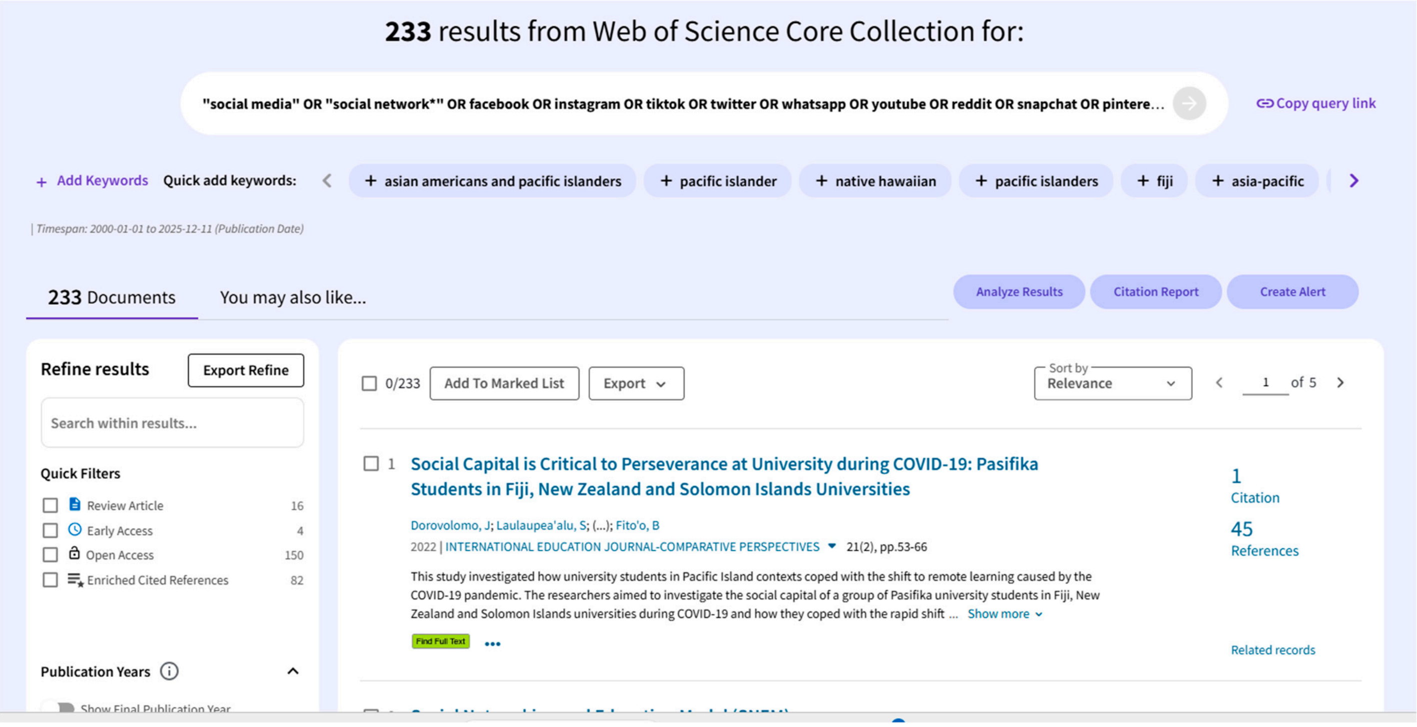

Grey literature searches

<table>
<tr><td colspan="2">Searches using the keywords “Pacific”, “social media”, “health”

Google search – first 50 results
Governmental and health ministry websites

American Samoa
Australia
Cook Islands
Federated States of Micronesia
Fiji
Guam
Hawaii
Kiribati
Marshall Islands
Nauru
New Caledonia
New Zealand
Palau
Papua New Guinea
Samoa
Solomon Islands
Tahiti/French Polynesia
Tokelau
Tonga
Tuvalu
United States US Department of Health and Human Services
Vanuatu

Organisation websites

World Health Organization
LeVa
Moana Connect
Pacific Community (SPC)

</td></tr>
<tr><td>Proquest for thesis repositories</td><td>abstract(Pacific OR Pasifika OR Tonga OR Samoa OR Tuvalu OR Fiji OR Cook Island OR Niue OR Tokelau OR Kiribati OR Nauru OR Marshall Island OR New Caledonia OR Palau OR Guam OR Tahiti OR Hawaii OR Solomon Island OR Vanuatu OR Wallis and Futuna OR Papua New Guinea OR PNG OR French Polynesia OR Melanesia OR Micronesia OR Polynesia) AND abstract(social media OR facebook OR instagram OR tiktok OR twitter OR whatsapp OR youtube OR reddit OR snapchat OR pinterest OR linkedin) AND abstract(health OR wellbeing OR well-being OR well being)</td></tr>
</table>
